# Effects of Small Peptide Supplementation on Growth Performance, Intestinal Barrier of Laying Hens During the Brooding and Growing Periods

**DOI:** 10.3389/fimmu.2022.925256

**Published:** 2022-07-07

**Authors:** Xiyu Zhao, Yao Zhang, Wentao He, Yuanhang Wei, Shunshun Han, Lu Xia, Bo Tan, Jie Yu, Houyang Kang, Mengen Ma, Qing Zhu, Huadong Yin, Can Cui

**Affiliations:** ^1^ Farm Animal Genetic Resources Exploration and Innovation Key Laboratory of Sichuan Province, Sichuan Agricultural University, Chengdu, China; ^2^ College of Forestry, Sichuan Agricultural University, Chengdu, China; ^3^ Key Laboratory for Animal Disease Resistance Nutrition of China, Institute of Animal Nutrition, Ministry of Education, Sichuan Agricultural University, Chengdu, China; ^4^ Triticeae Research Institute, Sichuan Agricultural University, Chengdu, China; ^5^ College of Resources, Sichuan Agricultural University, Chengdu, China

**Keywords:** small peptide, laying hen, growth performance, immune, anti-oxidation, intestinal health

## Abstract

The growing period is a critical period for growth and development in laying hens. During this period, chicks grow rapidly, but are accompanied by unstable digestive function, incomplete organ development, and high mortality. Small peptide, a feed additive, which has been proved to promote intestinal development and immunity in poultry. In order to elucidate the effects of small peptides on growth performance, immunity, antioxidant capacity, and intestinal health of growing laying hens, a total of 900 Tianfu green shell laying hens (1-day-old) were randomly divided into 5 treatments with 6 replicates of 30 birds each in this 18-week trial. Dietary treatments included a corn-soybean meal-based diet supplemented with 0 g/kg, 1.5 g/kg, 3.0 g/kg, 4.5 g/kg and 6.0 g/kg small peptide, respectively. The results showed that the supplementation of small peptides significantly increased growth rate (*P*<0.05) in laying hens, as well as elevated the serum immunoglobulins (*P*<0.05) and antioxidant indices (*P*<0.05), however, it decreased inflammation parameters (*P*<0.05). The supplementation of small peptides enhanced the intestinal function by promoting gut development (*P*<0.05) and improving gut integrity (*P*<0.05), barrier function (*P*<0.05) and the diversity of gut microbiota (*P*<0.05) in the growing hens. The best performance was recorded among the hens fed 4.5 g/kg level of small peptide. Taken together, these results showed that small peptide supplementation could improve the economic value of growing hens by promoting growth rate, disease resistance, and the optimal amount of addition for Tianfu green shell laying hens was 4.5 g/kg.

## Introduction

In chicken, brooding and growing periods are the most important stages in chicken breeding. However, these stages are characterized with many health problems such as bone disorder, loss of body weight, and abnormal faeces. This could be attributed to intestinal damage, which eventually reduced absorption in the chicken.

The ban on commercial antibiotics usage in the animal industry causes researchers to develop alternate feed supplements. Recently, feed additives such as amino acids, trace elements, and vitamins (1–3), as well as probiotics, polyphenols, and flavonoid have been utilized to maximize poultry immunity and productivity (4–6).

Small peptides are final products obtained from protein digestion in the gastrointestinal tract. They exhibit many biological functions in animals (7). They generally consist of 2-3 amino acids with an average molecular weight of approximately 300 daltons. Animals require certain concentration of small peptides to achieve optimal performance. Studies have reported small peptides promoted intestinal growth and development by improving intestinal structure and function, thereby promoting nutrient absorption and assimilation (8). Furthermore, some small molecules of active peptides were also reported to act on immune cells, thereby enhancing immunity and disease resistance in animals (9). It is therefore important to supplement chicken diets with small peptides because it provides physiological need for laying hens, as well as promotes growth and reduces mortality.

Previous studies on small peptides mainly focused on broiler breeders and laying hens during the laying period (10, 11). However, promoting the health and growth of laying hens during the brooding period is important to enhance production performance in hens. Furthermore, we hypothesized that small peptides may have important nutritional and regulatory functions on the growth of laying hens. Therefore, the aim of this study was to evaluate the effects of different doses of small peptides on growth performance, immune function, antioxidant capacity, and intestinal structure and barrier integrity, as well as gut microbiota composition of laying hens.

## Materials and Methods

### Experimental Design

A total of 900 1-week-old Tianfu green shell laying hens were assigned according to the body weight and then randomly allocated to five groups with six replicates for each group and thirty hens per replicate. Each of the treatment groups was fed a basal diet supplemented with different dose of small molecular plant active peptide (1.5 g/kg, 3.0 g/kg, 4.5 g/kg or 6.0 g/kg), except for the control group, which was fed a basal diet. And the basal diet was formulated to meet the recommended nutrient content by the National Research Council (1994) and was shown in **Table 1**. The small peptide product used in this experiment was produced by Mytech Company (Chengdu, China) with dehulled soybean meal (SBM) and soybean protein concentrate (SPC) as raw materials by combined liquid enzymatic hydrolysis process. And the molecular weight distribution was shown in **Table S1**. The experiment lasted for 18 weeks, and the birds were raised from 1-day till they reached egg production stage.

**Table 1:** Compositions and nutrient levels of the basal diets.

Raw materials (%)	Amounts	Nutrient levels (%)	Amounts
Corn grain	62.00%	AMEn^2^ (kcal/kg)	2677.99
Soybran bran	20.40%	Crude protein (CP)	16.36%
Wheat bran	3.00%	Available phosphorus	0.32%
Calcitic limestone	9.82%	Calcium	4.05%
Meat and bone meal (40% CP)	4.00%	Sodium	0.18%
Salt	0.38%	Digestible lysine	0.75%
Vitamin and mineral supplement^1^	0.20%	Digestible Met+Cys	0.59%
DL-methionine	0.14%	Digestible methionine	0.37%
L-Lysine	0.04%	Digestible threonine	0.54%

^1^Provided per kilogram of diets: VA 8000 IU, VD_3_ 1600 IU, VE 70 IU, VK_3_ 4 mg, VB_1_ 35 mg, VB_2_ 12 mg,

VB_6_ 7 mg, VB_12_ 0.2 mg, D-pantothenate 16 mg, Biotin 4 mg, niacin acid 60 mg, VC 200 mg. Cu (as copper sulfate) 20 mg, Fe (as ferrous sulfate) 83 mg, Mn (as manganese sulfate) 314 mg, Zn (as zinc sulfate) 174 mg, I (as potassium iodide) 13 mg, Se (as sodium selenite) 44 mg.

^2^AMEn—Nitrogen-corrected apparent metabolizable energy.

### Growth Performance Measurement

The body weight, tibia length, and feed intake of all chickens were measured weekly. The uniformity of body weight was calculated according to the following formula. The body weight was measured using the electronic balance, whereas the tibia length was measured using vernier caliper. Moreover, the feed intake was measured on per replicate basis.


Weight uniformity (%)=Birds in standard weight range/total birds ×100%

Standard weight lower limit=average weight−10% average weight

Standard weight upper limit=average weight+10% average weight

### Serum Collection and Indicator Determination

At the end of week 18, one chicken was randomly selected from each replicate per group, and blood samples were collected from the wing vein. The blood samples were centrifuged at 3000 rpm for 15 minutes, thereafter the serum was collected and stored at -80°C for subsequent analyses.

The serum indices including Immunoglobulin A (IgA), Immunoglobulin M (IgM), Immunoglobulin G (IgG), Interleukin-1β (IL-1β), Interleukin-6 (IL-6), Interleukin-8 (IL-8), Interleukin-12 (IL-12), Total antioxidant capacity (T-AOC), Superoxide dismutase (SOD), Glutathione peroxidase (GSH-Px), Malondialdehyde (MDA) were measured using ELISA kits according to the manufacturer’s instructions (MEIMIAN, Yancheng, China).

### Intestinal Tissue Samples Collection

At the end of week 18, 3 chickens were randomly selected from each group and euthanized. Thereafter, the small intestinal segments (duodenum, jejunum and ileum) were collected. Each section of the intestinal segment was divided into two parts: one part was stored in 4% paraformaldehyde fixative solution for subsequent tissue sectioning, and the other part was placed in liquid nitrogen and stored at -80°C for tissue RNA extraction. Finally, cecal contents were collected into cryopreservation tubes for microbial 16S rRNA sequencing.

### Intestinal Histomorphology

Paraffin sections were made with fixed intestinal segments, stained with hematoxylin-eosin, and then was observed and photographed under an electronic microscope. Image Pro Plus (IPP) was used to determine the intestinal characteristics such as villi height and crypt depth, and then the ratio of villus height to crypt depth (V/C) was calculated.

### Intestinal Tissue RNA Extraction and Quantitative Real-Time Polymerase Chain Reaction (qPCR)

The intestinal tissue samples were grounded into powder in a high-temperature sterilized mortar, and then total RNA was extracted from the intestinal tissues using trizol reagent (Takara, Dalian, China).

Reverse transcription of the RNA samples with the PrimeScript^®^ RT reagent Kit (Takara, Dalian, China), and the qPCR was used to detect the transcription levels of the tight junction proteins, immune factors and antioxidant related genes. The reaction system includes 5 µL SsoFastTM EvaGreen^®^ supermix (Takara, Dalian, China), 0.5µl Forward Primer, 0.5 µL Reverse Primer, 3 µL RNase Free H_2_O, and 1 µL cDNA sample. Relative gene expression was calculated using the 2^-△△CT^ method. The primers used for the quantitative real-time PCR is presented in **Table S2**.

### Caecal Microbial Sequencing Analysis

Total microbial genomic DNA was extracted using the Cetyltrimethylammonium Bromide (CTAB) method, and then the V3~V4 regions of the bacterial 16S rRNA gene were amplified by Polymerase Chain Reaction (PCR). The PCR products were confirmed with 2% agarose gel electrophoresis. Then, the products were purified by AMPure XT beads (Beckman Coulter Genomics, Danvers, MA, USA) and quantified by Qubit (Invitrogen, USA). The Beijing Novogene Technology Co. Ltd subsequently performed the sequencing and analysis.

### Statistical Analysis

Statistical analysis was performed using SPSS 19.0 statistical software (SPSS Inc., Chicago, IL). The analysis method was one-way ANOVA, and use the linear and quadratic effects to analyze the data. All the data were presented as the least-squares Mean and SEM. Statistical significance was set at *P*<0.05.

## Results

### Effects of Small Peptide Supplementation on Growth Performance

The weekly weight and uniformity of laying hens in each group were shown in **Tables 2** and **3**. Starting from 3 week of age and with increasing age, the weight of chickens gradually increased in the experimental groups fed diets with small peptides compared with the control group (*P*<0.05). At the 18^th^ week of age, the weight of the chicken in the supplemental groups was higher than that of the control group by more than 100g (*P*<0.05). Dietary small peptides addition had a quadratic effect (*P_Q_* <0.05) on the weight and uniformity of the chickens and the results showed that the 4.5 g/kg group had the most significant effect. In addition, the weight uniformity of the chickens in the supplemental groups also improved accordingly.

**Table 2:** Effects of small peptides on body weight of chickens.

Week	Groups					SEM	P-values	
	0 g/kg	1.5 g/kg	3.0 g/kg	4.5 g/kg	6.0 g/kg		Linear	Quadratic
0	34.38	34.87	34.55	33.93	34.38	2.02	0.462	0.787
3	134.92^a^	143.34^ab^	149.72b	148.84^b^	146.24^b^	18.84	0.147	0.012
6	299.24^a^	336.80^b^	336.72^bc^	337.40^bc^	319.16^ac^	32.64	0.526	0.074
9	573.80^a^	623.20^b^	627.08^b^	641.00^b^	613.80^ab^	55.52	0.276	0.064
12	789.00^a^	874.90^b^	883.10^b^	892.70^b^	871.80^b^	68.52	0.193	0.057
15	981.20^a^	1065.90^ab^	1082.70^b^	1094.90^b^	1082.20^b^	81.13	0.109	0.034
18	1175.00^a^	1290.50^b^	1303.00^b^	1318.50^b^	1278.00^b^	100.28	0.234	0.047

^abc^Mean values in the same row having different superscript letters differed significantly (P<0.05).

**Table 3:** Effects of small peptides on weight uniformity of chickens.

Week	Groups					P-values	
	0 g/kg	1.5 g/kg	3.0 g/kg	4.5 g/kg	6.0 g/kg	Linear	Quadratic
0	100%	96%	100%	100%	100%	0.559	0.786
3	52%	64%	72%	58%	72%	0.548	0.492
6	56%	72%	76%	76%	60%	0.744	0.021
9	56%	68%	88%	88%	48%	0.956	0.174
12	72%	80%	80%	90%	70%	0.873	0.133
15	80%	80%	90%	90%	70%	0.761	0.327
18	60%	80%	90%	90%	70%	0.547	0.017

The tibia length of each treatment group was shown in **Table 4**. For the tibia length, we observed no significant differences within the first three weeks of age among the experimental groups (*P*<0.05). Moreover, from the 6^th^ week, the tibia length of the supplemental groups increased significantly compared with the control group (*P*<0.05). But there were no linear and quadratic effects among experimental treatments for tibia length (*P_L_* >0.05, *P_Q_* >0.05).

**Table 4:** Effects of small peptides on tibia length of chickens.

Week	Groups					SEM	P-values	
	0 g/kg	1.5 g/kg	3.0 g/kg	4.5 g/kg	6.0 g/kg		Linear	Quadratic
0	26.84	26.08	28.26	28.03	27.82	0.92	0.212	0.510
3	48.00	48.84	49.77	48.40	48.68	1.89	0.721	0.272
6	63.28^a^	65.85^b^	64.15^a^	64.17^a^	63.52^a^	2.79	0.761	0.570
9	75.15^a^	77.84^b^	79.98^c^	78.12^bc^	77.22^ab^	3.76	0.503	0.102
12	96.43^a^	98.63^b^	98.18^b^	98.22^b^	99.69^b^	3.30	0.088	0.313

^abc^Mean values in the same row having different superscript letters differed significantly (P<0.05).

**Tables 5** and **6** summarized the results of the average daily feed intake (ADFI) and average daily gain (ADG) of the chickens. No significant difference was observed for the feed intake among all the experimental groups (*P*>0.05). However, from the sixth week, the ADG of the supplemental groups was significantly higher than that of the control group (*P*<0.05). In addition, small peptides improved ADG in a quadratic manner (*P_Q_* <0.05).

**Table 5:** Effects of small peptides on the ADFI of chickens.

Week	Groups					SEM	P-values	
	0 g/kg	1.5 g/kg	3.0 g/kg	4.5 g/kg	6.0 g/kg		Linear	Quadratic
3	11.67	11.27	11.56	11.18	11.29	4.01	0.244	0.539
6	24.04	24.25	24.46	24.58	23.58	6.56	0.702	0.222
9	43.00	43.64	43.72	43.59	41.66	6.21	0.392	0.069
12	61.10	60.74	61.14	61.72	61.24	5.33	0.320	0.680
15	73.52	73.94	73.89	73.98	74.42	3.38	0.033	0.169
18	83.18	83.21	83.12	83.25	83.18	3.79	0.831	0.979

**Table 6:** Effects of small peptides on the ADG of chickens.

Week	Groups					SEM	P-values	
	0 g/kg	1.5 g/kg	3.0 g/kg	4.5 g/kg	6.0 g/kg		Linear	Quadratic
3	4.28	5.27	5.51	5.50	5.45	0.53	0.124	0.047
6	7.83^a^	8.99^ab^	9.41^b^	9.10^b^	8.43^ab^	0.46	0.586	0.010
9	13.11^a^	13.70^ab^	13.92^ab^	14.59^b^	14.17^ab^	0.44	0.059	0.108
12	10.24^a^	12.07^b^	12.27^b^	12.19^b^	12.29^b^	0.47	0.140	0.109
15	9.08^a^	9.19^a^	9.64^ab^	9.98^b^	10.03^b^	0.28	0.006	0.050
18	9.13^a^	9.22^a^	10.45^ab^	10.67^b^	9.37^ab^	0.47	0.485	0.348

^abc^Mean values in the same row having different superscript letters differed significantly (P<0.05).

### Effects of Small Peptide supplementation on Serum Immune Levels

**Figure 1:**
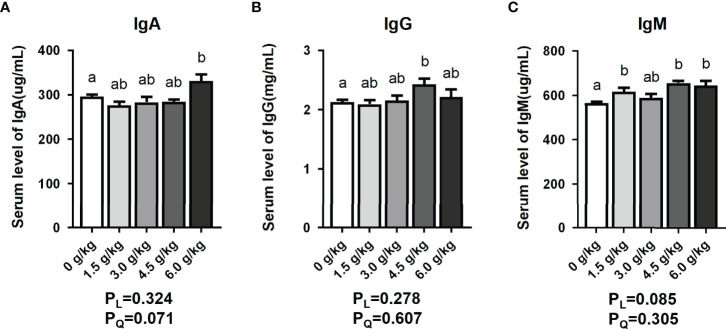
Effects of small peptide supplementation on Serum Immune Levels of laying hens at 18 weeks of age. **(A)** Effects of different concentrations of small peptides on the serum IgA levels. **(B)** Effects of different concentrations of small peptides on the serum IgG levels. **(C)** Effects of different concentrations of small peptides on serum IgM levels. Data represent the mean ± SEM (n=6 independent samples). Bars having different superscript letters differed significantly (*P*<0.05).

In the **Figure 1** of this study, the serum IgA content of the 6.0 g/kg group increased significantly (*P*<0.05), whereas the serum IgG content of the 4.5 g/kg group was significantly higher than that of the control group (*P*<0.05). Moreover, serum IgM levels of the 1.5 g/kg, 4.5 g/kg, and 6.0 g/kg groups showed a significant upward trend (*P*<0.05).

### Effects of Small Peptide supplementation on Serum Inflammatory Cytokines

**Figure 2:**
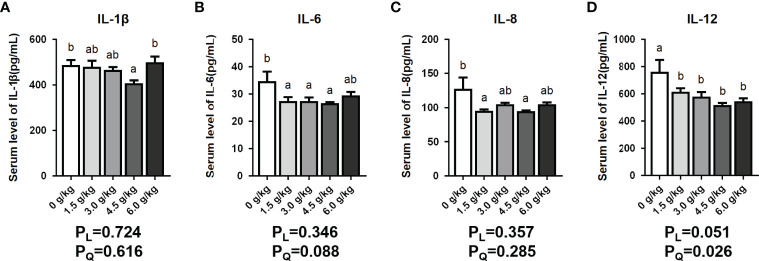
Effects of small peptide supplementation on serum inflammatory levels of laying hens at 18 weeks of age. **(A)** Effects of different concentrations of small peptides on the level of IL-1β in the serum. **(B)** Effects of different concentrations of small peptides on the level of IL-6 in the serum. **(C)** Effects of different concentrations of small peptides on the level of IL-8 in the serum. **(D)** Effects of different concentrations of small peptides on the level of IL-12 in the serum. Data represent the mean ± SEM (n=6 independent samples). Bars having different superscript letters differed significantly (*P*<0.05).

In this present study, the serum interleukin levels presented in **Figure 2** showed that the levels of serum IL-6, IL-8, and IL-12 were significantly reduced in the chicken group fed the supplementation of 1.5 g/kg small peptide (*P*<0.05), and the 3.0 g/kg group showed a downward trend (*P*<0.05). Moreover, the 4.5 g/kg group showed the best anti-inflammatory effect, and all four interleukins were significantly reduced (*P*<0.05). In the 6.0 g/kg group, only the serum level of IL-12 decreased significantly (*P*<0.05). Dietary small peptides addition had a quadratic effect (*P_Q_* <0.05) on the serum levels of IL-1β and IL-12.

### Effects of Small Peptide Supplementation on Serum Antioxidant Index Levels

**Figure 3:**
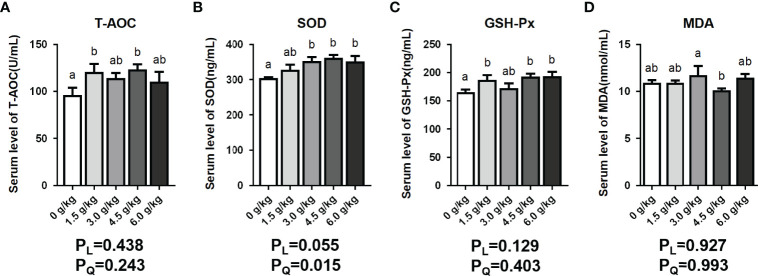
Effects of small peptide supplementation on serum antioxidant index levels of laying hens at 18 weeks of age. **(A)** Effects of different concentrations of small peptides on the level of T-AOC in serum. **(B)** Effects of different concentrations of small peptides on the level of SOD in serum. **(C)** Effects of different concentrations of small peptides on the level of GSH-Px in serum. **(D)** Effects of different concentrations of small peptides on the level of MDA in the serum. Data represent the mean ± SEM (n=6 independent samples). Bars having different superscript letters differed significantly (*P*<0.05).

It was observed in **Figure 3** of this present study that the serum GSH-Px levels of the chickens in the 1.5 g/kg group, 4.5 g/kg group, and 6.0 g/kg group increased significantly (*P*<0.05). In addition, the serum SOD levels of the 3.0 g/kg group, 4.5 g/kg group, and 6.0 g/kg group were elevated significantly (*P*<0.05). Moreover, the serum T-AOC levels of the supplemental groups were enhanced to varying degrees, however, the 1.5 g/kg and 4.5 g/kg groups recorded significant differences (*P*<0.05). Furthermore, the 4.5 g/kg decreased the levels of serum MDA content (*P*<0.05). Results showed a quadratic increase (*P_Q_* <0.05) in serum SOD content of birds fed small peptides diets.

### Effects of Small Peptide Supplementation on Intestinal Morphology of Chickens

**Figure 4:**
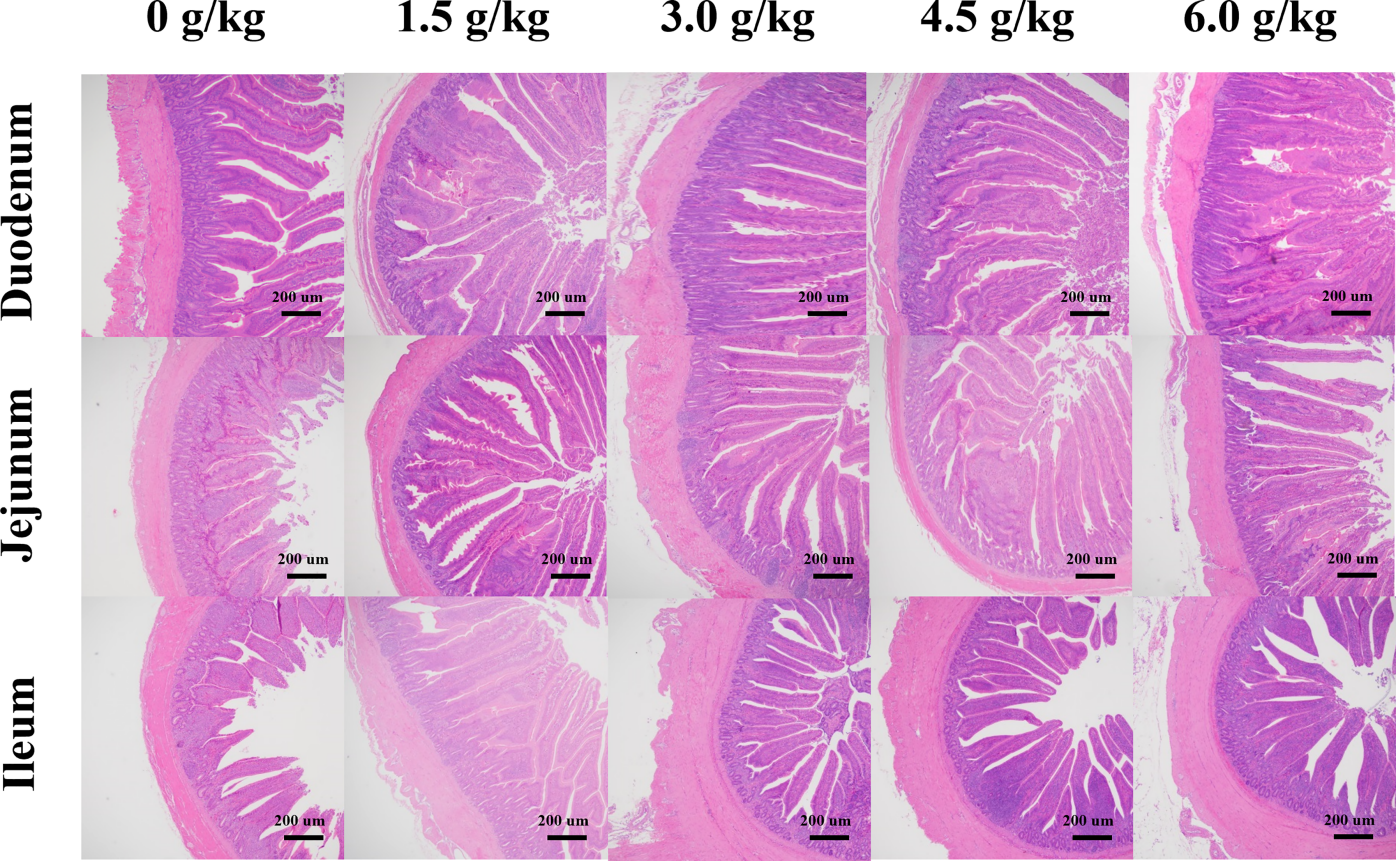
Effects of small peptide supplementation on the small intestinal morphology of laying hens at 18 weeks of age. The villus length and crypt depth were observed in sections of different intestinal segments (40×).

The results obtained in the present study showed that (**Table 7** and **Figure 4**), the supplementation of small peptides significantly increased the ratio of the villi height and crypt depth (*P*<0.05), where 4.5 g/kg was the most appropriate concentration compared with the other groups. And small peptides improved the villus length and V/C of Jejunum in a quadratic manner (*P_Q_* <0.05).

**Table 7:** Effects of small peptide on small intestinal morphology.

Position	Indicator	0 g/kg	1.5 g/kg	3.0 g/kg	4.5 g/kg	6.0 g/kg	SEM	Linear	Quadratic
Ileum	Villus length	984.42^a^	1054.20^b^	1057.44^b^	1244.47^c^	1206.33^c^	36.18	0.034	0.178
	Crypt depth	206.08^a^	175.98^b^	173.18^b^	201.15^a^	193.44^a^	8.46	0.840	0.619
	V/C^1^	4.79^a^	5.98^c^	6.11^b^	6.19^b^	6.23^bc^	0.18	0.099	0.081
Jejunum	Villus length	732.90^a^	931.77^b^	1019.50^c^	1057.18^c^	891.63^b^	23.27	0.335	0.023
	Crypt depth	206.08^a^	208.60^a^	204.12^a^	211.60^a^	174.96^b^	6.13	0.254	0.247
	V/C^1^	3.55^a^	4.46^b^	5.00^c^	5.00^c^	5.10^c^	0.19	0.046	0.019
Duodenum	Villus length	684.11^a^	788.41^b^	644.35^a^	763.13^b^	1019.24^c^	24.55	0.189	0.243
	Crypt depth	167.41^b^	198.81^c^	134.91^a^	138.32^a^	242.47^d^	9.41	0.606	0.503
	V/C^1^	4.08^a^	3.96^a^	4.78^b^	5.54^c^	4.22^a^	1.61	0.447	0.544

^abc^Mean values in the same row having different superscript letters differed significantly (P<0.05).

V/C^1^ = villi height/crypt depth.

Each value represents the mean of six replicates (one bird per replicate, 10 readings per bird).

### Effects of Small Peptide Addition on Intestinal Tight Junction Protein Expression

**Figure 5:**
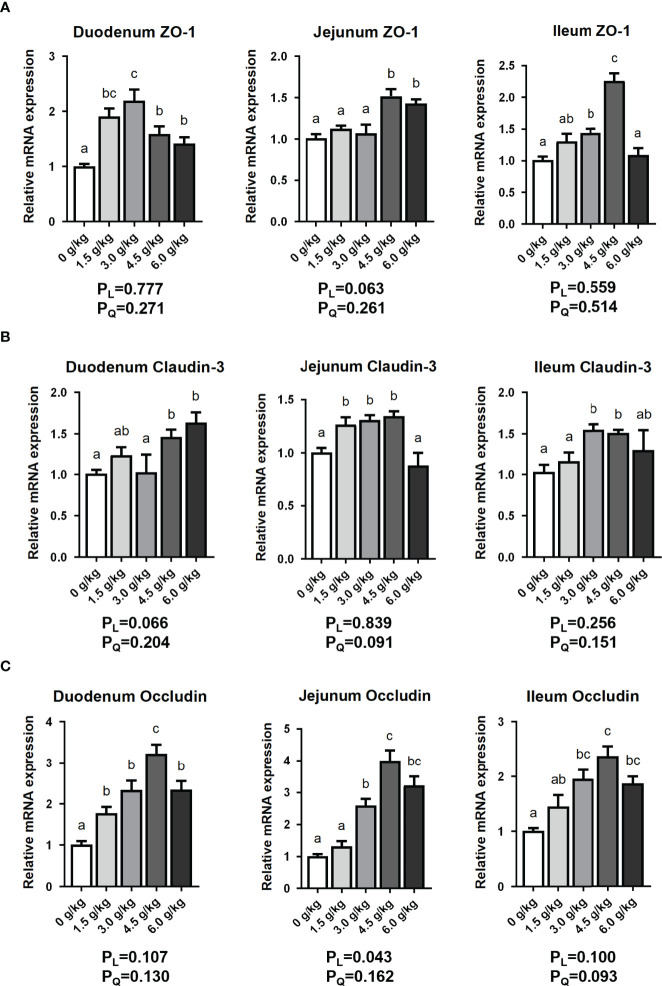
Effects of small peptide supplementation on the expression of intestinal tight junction protein of laying hens at 18 weeks of age. **(A)** Effects of different concentrations of small peptides on the expression of *ZO-1* mRNA of the small intestinal segments (duodenum, jejunum, and ileum). **(B)** Effects of different concentrations of small peptides on the expression of *Claudin-3* mRNA of the small intestinal segments (duodenum, jejunum and ileum). **(C)** Effects of different concentrations of small peptides on the expression of *Occludin* mRNA of the small intestinal segments (duodenum, jejunum and ileum). Data represent the mean ± SEM (n=3 independent samples). Bars having different superscript letters differed significantly (*P*<0.05).

As shown in **Figure 5**, we found that the mRNA expression levels of Zonula occludin-1 (*ZO-1*), *Claudin-3* and *Occludin* in the duodenum, *Claudin-3* in the jejunum and *ZO-1* in the ileum of chickens in the 1.5 g/kg group were significantly upregulated (*P*<0.05). In the 3.0 g/kg group, the expression levels of duodenum *ZO-1* and *Occludin*, jejunum *Claudin-3* and *Occludin*, ileum *ZO-1*,*Claudin-3* and *Occludin* increased significantly (*P*<0.05). The 4.5 g/kg group recorded the best results, and the expression of three tight junction proteins in the three small intestinal segments all increased significantly (*P*<0.05). Furthermore, the levels of expression *ZO-1*, *Claudin-3* and *Occludin* in the duodenum, *ZO-1* and *Occludin* in the jejunum, and *Occludin* in the ileum were significant increased in the 6.0 g/kg group (*P*<0.05). However, there were no linear and quadratic effects among experimental treatments for intestinal tight junction protein expression (*P_L_* >0.05, *P_Q_* >0.05).

### Effects of Small Peptide Supplementation on the Expression of Intestinal Immune Factors

**Figure 6:**
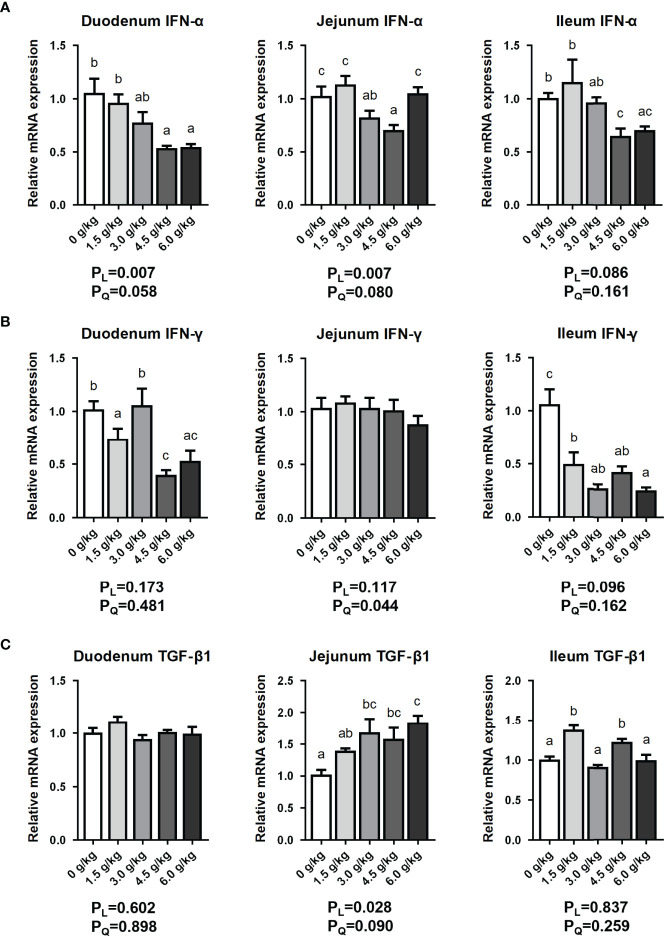
Effects of small peptide supplementation on the intestinal immune factors expression of laying hens at 18 weeks of age. **(A)** Effects of different concentrations of small peptides on the expression of *IFN-α* mRNA in each intestinal segment (duodenum, jejunum and ileum). **(B)** Effects of different concentrations of small peptides on the expression of *IFN-γ* mRNA in each intestine (duodenum, jejunum and ileum). **(C)** Effects of different concentrations of small peptides on the expression of *TGF-β1* mRNA in each intestine (duodenum, jejunum and ileum). Data represent the mean ± SEM (n=3 independent samples). Bars having different superscript letters differed significantly (*P*<0.05).

In this present study, as shown in **Figure 6**, the expression of *Interferon-alpha* (*IFN-α*) mRNA in duodenal segment was significantly decreased by the supplementation of 4.5 g/kg and 6.0 g/kg compared with the control and 1.5 g/kg groups (*P*<0.05). Moreover, in the jejunal segment, the mRNA expression of *IFN-α* was significantly decreased by the supplementation of 3.0 g/kg and 4.5 g/kg small peptides compared with the other groups (*P*<0.05). Furthermore, in the ileal segment, the expression of *IFN-α* mRNA was significantly decreased by the supplementation of 4.5 g/kg and 6.0 g/kg compared with the other groups (*P*<0.05). In terms of *Interferon-gamma* (*IFN-γ*), the experimental group had a significant down-regulation effect in the duodenum and ileum compared with the control group. In addition, feeding small peptides significantly enhanced the expression of *transforming growth factor-β1* (*TGF-β1*) in the jejunum and ileum of the chickens. Small peptides supplementation influenced intestinal *IFN-α* and *TGF-β1* mRNA expressions in a dose-dependent manner (*P_L_* <0.05), and decreased *IFN-γ* mRNA expression in a quadratic manner (*P_Q_* <0.05).

### Effects of Small Peptide Supplementation on the Intestinal Antioxidant Capacity

**Figure 7:**
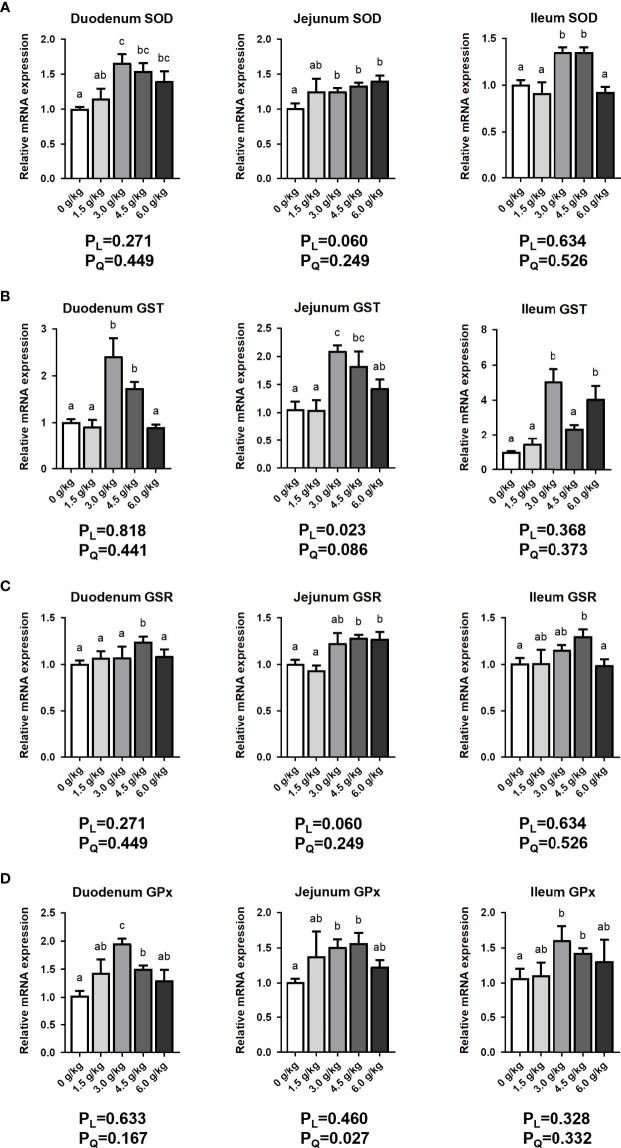
Effects of small peptide supplementation on the intestinal Antioxidant capacity of laying hens at 18 weeks of age. **(A)** Effects of different concentrations of small peptides on the expression of *SOD* mRNA in each intestinal segment (duodenum, jejunum, and ileum). **(B)** Effects of different concentrations of small peptides on the expression of *GST* mRNA in each intestinal (duodenum, jejunum, and ileum). **(C)** Effects of different concentrations of small peptides on the expression of *GSR* mRNA in each intestinal segment (duodenum, jejunum, and ileum). **(D)** Effects of different concentrations of small peptides on the expression of *GPx* mRNA in each intestine (duodenum, jejunum, and ileum). Data represent the mean ± SEM (n=3 independent samples). Bars having different superscript letters differed significantly (*P*<0.05).

**Figure 7** in this study showed that different levels of small peptides significantly increased the mRNA expressions of *SOD*, *Glutathione S-transferase* (*GST*), *Glutathione Reductase* (*GSR*), and *Glutathione Peroxidase* (*GPx*) in the duodenum, jejunum, and ileum of the laying hens (*P*<0.05). In addition, the effect of small peptide addition on jejunum GST mRNA expression showed a linear effect (*P_L_* <0.05), and the expression of GPx mRNA showed a quadratic effect (*P_Q_* <0.05).

### Effects of Small Peptide Supplementation on Cecal Microbiota Composition

**Figure 8:**
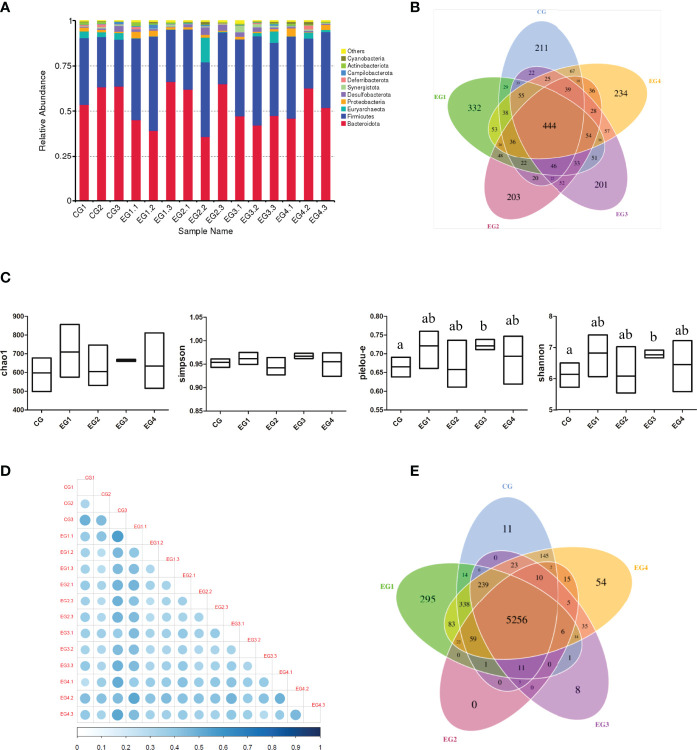
Effects of small peptide supplementation on cecal microbial diversity of laying Hens at 18 weeks of age. **(A)** Histogram of relative abundance top10 of species at phylum level. **(B)** The petal map representing common and unique ASVs among each group. **(C)** Alpha diversity indexes (chao1, simpson, pielou-e and shannon) for each group. **(D)** The heat map of Beta diversity index Unweighted unifrac distance matrix: The larger the circle is, the darker the corresponding color is, indicating the greater the difference between the two samples. On the contrary, the smaller the circle, the lighter the corresponding color, indicating that the difference between the two samples is smaller. **(E)** Function annotation petal diagram: The common and specific gene information among different groups was analyzed, and the results of KO database function prediction were displayed.

**Figure 8A** represented the relative abundance of the top 10 bacteria species with the largest abundance at the phylum level in each group of cecal content samples. Compared with the control group, the abundance of *firmicutes* in the four experimental groups increased, whereas the abundance of *Bacteroidetes* decreased. Moreover, **Figure 8B** showed that the Amplicon Sequence Variants (ASVs) was unique among the various groups. The results showed that the 1.5 g/kg group had 121 more specific ASVs than the control group. In addition, **Figure 8C** showed the Alpha diversity indexes, and it could be seen that these Alpha diversity indexes in the 4.5 g/kg group were significantly higher than those in the control group. Through the beta diversity analysis of each sample, we found that CG3 (Control group, 0 g/kg group), EG1.1 (experimental group 1, 1.5 g/kg group) and EG4.2 (experimental group 4, 6.0 g/kg group) are significantly different from the other samples (**Figure 8D**). Subsequently, we performed function prediction based on KO database for each of the groups per sample sequencing data, and the results showed that there were 5256 common functions among the five groups, and the number of unique functions in the 1.5 g/kg group was more than 284 in the control group (**Figure 8E**).

## Discussion

Most of the final products of protein digestion in the digestive tract are small peptides, and small peptides can enter the systemic circulation through intestinal mucosal cells (12). Studies have shown that small peptides and amino acids have independent absorption mechanisms, and they do not interfere with each other, and the small peptides themselves can promote the transport of other amino acids or peptides, which can alleviate the absorption competition between amino acids (13). The nutritional effect of small peptides is reflected in the growth performance at the individual level. Karimzadeh showed that addition of Canola Bioactive Peptides (CBP) at 200 and 250 g/kg diet improved body weight gain (BWG) and decreased the feed to gain ratio (F/G) of broilers (14). Abdollahi showed that soybean bioactive peptides (SBP) had a significant effect on the F/G of broilers (15). Consistent with these research studies, in this study, the weight and uniformity of chickens increased significantly in the experimental groups fed diets with small peptides compared with the control group (*P*<0.05). In addition, a significant effect of small peptides was observed for the ADG of chicks (*P*<0.05). Tibia is an important organ for the growth and production of laying hens. The development of tibia before laying plays an important role in laying performance (16). Some small peptides have the property of binding to metals, which can promote the passive transport process and storage of metal elements in the body (17). Casein phosphopeptide (CPP) can combine with calcium in the small intestine of animals to prevent the formation of calcium phosphate precipitation, which greatly increases the content of dissolved calcium in the intestine, thereby promoting the absorption and utilization of calcium (18). In this study, dietary small peptides supplementation increased tibia length of chickens (*P*<0.05), which may be due to the increased absorption and utilization of minerals, especially calcium and phosphorus.

Immunoglobulins are important components of the humoral immunity, which form a basis for the body’s anti-infection immunity (19). The immunoglobulin levels reflect the immune capacity of animals. IgA is the main component of the mucosal defense system which is responsible for inhibiting microbial attachment, hence preventing pathogenic bacteria effection, thereby improving immune barrier function (20). IgM is the immunoglobulin with the largest molecular weight. It has bactericidal, complement system activation, immune regulation and agglutination effects, and is the body’s first line of defense (21). IgG is the main component of immunoglobulin in the serum which has antiviral, neutralizing virus, antibacterial, and immune regulatory properties (22). In this study, small peptide supplementation can significantly improve the serum immunoglobulin levels of young chickens (*P*<0.05). Moreover, Studies have shown that soybean polypeptides contain antibodies that enhance animal immune function and improve animal health (23). Small peptides can participate in the immune regulation of the body and promote the phagocytosis of macrophages, lymphocyte and immature splenic cell proliferation which may be the reason for the increase of serum immune level (24).

Interleukin (IL) is a lymphoid factor that interacts between leukocytes or immune cells and its function is related to the expression and regulation of immune responses. IL-1β, also known as lymphocyte stimulator, plays an immunomodulatory role at low local concentrations (25). IL-6 stimulates B cell activation, T cell proliferation, and hepatocyte synthesis of acute phase proteins (26). IL-8 is responsible for attracting and activating neutrophils, as well as regulating local inflammatory responses (27). In addition, IL-12, produced by B cells and macrophages, acts on T cells and natural killer (NK) cells thereby promoting anti-tumor and anti-infection immunity (28). Therefore, IL-1β, IL-6, IL-8, and IL-12 are regarded as proinflammatory factors in immune response. Previous studies have shown that dietary SBP supplementation attenuated the coccidia challenge-induced effect on the expression of plasma IL-6, IL-10, and TNF-α. Our results showed that the dietary supplementation with the small peptides can reduce the levels of pro-inflammatory factors (*P*<0.05), thereby protecting the body from inflammatory damage. On the other hand, it may be related to the enhancement of immunity by small peptides.

Many internal and external factors such as breathing, external pollution, radiation and other factors contribute to production and accumulation of free radicals which eventually results in pre-mature aging and diseases among birds. Inert antioxidant enzymes resist free radicals from causing destruction to cells and tissues (29). GSH-Px is an important peroxidase enzyme which is play an important role in scavenging oxidative stress in animals. SOD is an antioxidant metal enzyme which catalyzes superoxide anion radical disproportionation to generate oxygen and hydrogen peroxide, and is a natural scavenger of oxygen free radicals in an organisms (30). T-AOC reflects the total ability of scavenging reactive oxygen species/nitric oxide synthase (ROS/NOS) to a certain extent. The content of MDA, the final product of lipid oxidation, is an important parameter reflecting the potential antioxidant capacity of the body, which can reflect the rate and intensity of lipid peroxidation and also indirectly reflect the degree of tissue peroxidation damage (31). Carnosine, an endogenous dipeptide composed of β -alanine and L-histidine, is another natural non-enzymatic free radical scavancer and antioxidant discovered after SOD and vitamin E, which can reduce cellular oxidative stress and inhibit the formation of intracellular reactive oxygen species and active nitrogen (32). Our data showed that different concentrations of small peptides can improve the antioxidant capacity of laying hens (*P*<0.05) by directly participating in the scavenging of free radicals *in vivo* or regulating the expression of antioxidant related enzymes. And it was observed in this study that 4.5 g/kg level of small peptide supplementation recorded the best results.

The small intestine is the largest digestive and absorptive organ in poultry. The epithelial structure of the small intestine is closely related to the digestion and absorption of nutrients. The intestinal villi height is positively correlated with the absorption of nutrients, whereas deep crypts depth indicates the maturity of intestinal epithelial cells. The lower the crypt depth the lower the nutrient absorption rate. Therefore, the ratio of the villi height to the crypt depth of the small intestine is an important indicator for measuring the health and absorption capacity of the intestines. Previous studies have shown that antimicrobial peptides, Fermented Soybean Meal (FSBM) and SBP significantly increase the V/C ratio (11, 15, 33). These are consistent with our results which indicated that small peptides can promote the growth and development of the intestinal tract (*P*<0.05) and ensure the integrity of intestinal structure and function, and improve the nutrient absorption and utilization rate of laying hens, and ultimately improve their growth performance.

The intestinal tract plays digestive and absorptive roles, as well as performs congenital barrier roles, thereby, maintaining stable internal environment (34). The intestinal barrier of poultry includes mechanical barrier, chemical barrier, microbial barrier, and immune barrier, and its integrity is closely related to intestinal function. The mechanical barrier, mainly composed of tight junction proteins. Tight junction proteins perform barrier functions by preventing the invasion of toxic macromolecules and microorganisms and also regulating the selective entry of small molecules and ions into the body (35). In addition, tight junction proteins are involved in the regulation of gene transcription, cell proliferation and differentiation. Tight junction proteins mainly include Claudin, Occludin and Zonula occludin family proteins (36). Our results showed that small peptides can promote the expression intestinal tight junction proteins (*P*<0.05) to maintain the integrity of the intestinal mucosal barrier and accelerate repair of its damage which is consistent with the experimental results of Osho *et al.* Their experiments showed that SBP reduced the coccidia induced effect on the expression of *ZO-1* and *ZO-2* (9).

Interferons (IFNs) are a group of signaling proteins synthesized and released by the host cells in response to invasion of pathogens such as viruses, bacteria, parasites, or tumor cells. Usually, virus-infected cells release interferon, which causes surrounding cells to improve their antiviral defenses (37). IFN-α and IFN-γ are involved in promoting innate immunity in response to viral infection. In addition, IFN-γ forms immune barrier to inhibit certain bacteria and protozoan infections, as well as activate macrophages (38). The expression levels of IFN-α and IFN-γ are negatively correlated with the intestinal inflammation and immunity. TGF-β plays an important role in the immune function of the body. TGF-β can inhibit the growth of many types of cells and induce the production of extracellular matrix, and is an angiogenesis-inducing factor. Also, TGF-β can antagonize many immune responses, including T cell and macrophage activation. It is also involved in inhibiting proliferation of immune active cells, as well as inhibits differentiation of lymphocytes and the production of inflammatory factors (39). Our results showed that small peptides can reduce the expression of pro-inflammatory factors (*P*<0.05) and increase the expression of anti-inflammatory factors (*P*<0.05) in the small intestine of laying hens, thereby improving intestinal immune level and promoting gut structure and integrity and barrier function. And 4.5 g/kg was the best treatment dose.

GST, GSR and GPx play important antioxidant functions in animals (40). Our results showed that small peptides can significantly increase the expression of various intestinal antioxidant stress-related genes (*P*<0.05) to reduce and repair the intestinal oxidative damage. 3.0 g/kg and 4.5 g/kg recorded the best results. Xie’s study had similar results, antimicrobial peptides can improve the antioxidant capacity of intestinal tract, increase the activity of SOD in intestinal tract, and decrease the content of MDA in intestinal tissue of broilers (11).

Intestinal microorganisms participate in many physiological and biochemical processes, such as host immune barrier, feed digestion, nutrient absorption, and energy utilization. Gut microbiota attains highest colonization during the brooding stage of chickens. The brooding stage in female chickens is the key stage for the transformation of digestive mode and rapid development of digestive tissue (41, 42). Therefore, we sequenced and analysed the gut microbes of chickens in the five groups of this experiment. Alpha diversity index reflects the diversity of microorganisms. The Chao1 index gives an estimation of the total number of species contained in the community sample, and the lower a bacteria abundance species in the community, the greater the Chao1 index. Pielou-e is the evenness index, and the more homogeneous the species, the greater the Pielou-e index. Shannon is used to measure the total number of categories and their proportions in each sample. The higher the community diversity, the more even the species distribution, and the larger the shannon index. Simpson is used to characterize the diversity and uniformity of bacteria specie distribution in the community. The better the species uniformity, the larger the Simpson index. Our results showed that these Alpha diversity indexes in the 4.5 g/kg group were significantly higher than those in the control group (*P*<0.05), which indicated that the supplementation of small peptides can affect the cecal microbiota composition and function in laying hens.

## Conclusion

In summary, the supplementation of different levels of small peptides during the brooding period improves the growth performance, immune function, and antioxidant capacity of laying hens. In addition, the supplementation of small peptides promotes intestinal structure and barrier integrity, as well as promotes gut microbiota composition, thereby improving gut health in birds. For Tianfu green shell hens, the best supplementation of small peptide in the basal diet is 4.5 g/kg.

## Data Availability Statement

The original contributions presented in the study are included in the article/**Supplementary Material**. Further inquiries can be directed to the corresponding author.

## Ethics Statement 

The animal study was reviewed and approved by Animal Care and Use Committee of Sichuan Agricultural University.

## Author Contributions

XZ, YZ, WH, and HY: conceptualization. XZ, YZ, and BT: formal analysis. HY and QZ: funding acquisition and writing – review and editing. XZ, YZ, WH, YW, and HK: investigation. XZ, SH, and JY: methodology. LX: project administration. WH: software. XZ, MM, and CC: validation. XZ: writing – original draft. All authors contributed to the article and approved the submitted version.

## Funding

This research was funded by The National Key Research and Development Program of China, grant number 2021YFD1300600; Sichuan Science and Technology Program, grant number 2021YFYZ0007 and 2022YFYZ0005, and China Agriculture Research System of MOF and MARA, grant number CARS-40.

## Conflict of Interest

The authors declare that the research was conducted in the absence of any commercial or financial relationships that could be construed as a potential conflict of interest.

## Publisher’s Note

All claims expressed in this article are solely those of the authors and do not necessarily represent those of their affiliated organizations, or those of the publisher, the editors and the reviewers. Any product that may be evaluated in this article, or claim that may be made by its manufacturer, is not guaranteed or endorsed by the publisher.

## References

[1] Kidd MT Maynard CW Mullenix GJ. Progress of Amino Acid Nutrition for Diet Protein Reduction in Poultry. J Anim Sci Biotechnol (2021) 12(1):45. doi: 10.1186/s40104-021-00568-0 33814010 PMC8020538

[2] Abd El-Hack ME Mahrose K Askar AA Alagawany M Arif M Saeed M. Single and Combined Impacts of Vitamin a and Selenium in Diet on Productive Performance, Egg Quality, and Some Blood Parameters of Laying Hens During Hot Season. Biol Trace Elem Res (2017) 177(1):169–79. doi: 10.1007/s12011-016-0862-5 27744603

[3] Hashem MA Abd El Hamied SS Ahmed EMA Amer SA El-Sharnouby ME. Mitigating the Growth, Biochemical Changes, Genotoxic and Pathological Effects of Copper Toxicity in Broiler Chickens by Supplementing Vitamins C and E. Anim (Basel) (2021) 11(6):1811. doi: 10.3390/ani11061811 PMC8234185 34204508

[4] Yuan ZH Zhang KY Ding XM Luo YH Bai SP Zeng QF. Effect of Tea Polyphenols on Production Performance, Egg Quality, and Hepatic Antioxidant Status of Laying Hens in Vanadium-Containing Diets. Poult Sci (2016) 95(7):1709–17. doi: 10.3382/ps/pew097 27044874

[5] Amevor FK Cui Z Ning Z Du X Jin N Shu G. Synergistic Effects of Quercetin and Vitamin E on Egg Production, Egg Quality, and Immunity in Aging Breeder Hens. Poult Sci (2021) 100(12):101481. doi: 10.1016/j.psj.2021.101481 34717121 PMC8564671

[6] Shokryazdan P Jahromi MF Md Saadand S Ebrahimi M Idrus Z Zhou H. Chinese Herbal Medicines as Potential Agents for Alleviation of Heat Stress in Poultry. Scientifica (Cairo) (2017) 2017:8208261. doi: 10.1155/2017/8208261 29209556 PMC5676482

[7] Salavati ME Rezaeipour V Abdullahpour R Mousavi N. Effects of Graded Inclusion of Bioactive Peptides Derived From Sesame Meal on the Growth Performance, Internal Organs, Gut Microbiota and Intestinal Morphology of Broiler Chickens. Int J Pept Res Ther (2020) 26(3):1541–8. doi: 10.1007/s10989-019-09947-8

[8] Kogut MH Genovese KJ He H Swaggerty CL Jiang Y. Modulation of Chicken Intestinal Immune Gene Expression by Small Cationic Peptides as Feed Additives During the First Week Posthatch. Clin Vaccine Immunol (2013) 20(9):1440–8. doi: 10.1128/cvi.00322-13 PMC3889598 23863505

[9] Osho SO Xiao WW Adeola O. Response of Broiler Chickens to Dietary Soybean Bioactive Peptide and Coccidia Challenge. Poult Sci (2019) 98(11):5669–78. doi: 10.3382/ps/pez346 31247645

[10] Li X Wei X Guo X Mi S Hua X Li N. Enhanced Growth Performance, Muscle Quality and Liver Health of Largemouth Bass (Micropterus Salmoides) Were Related to Dietary Small Peptides Supplementation. Aquaculture Nutrition (2020) 26(6):2169–77. doi: 10.1111/anu.13155

[11] Xie Z Zhao Q Wang H Wen L Li W Zhang X. Effects of Antibacterial Peptide Combinations on Growth Performance, Intestinal Health, and Immune Function of Broiler Chickens. Poult Sci (2020) 99(12):6481–92. doi: 10.1016/j.psj.2020.08.068 PMC7810918 33248563

[12] Sánchez A Vázquez A. Bioactive Peptides: A Review. Food Qual Saf (2017) 1(1):29–46. doi: 10.1093/fqsafe/fyx006

[13] Akbarian M Khani A Eghbalpour S Uversky VN. Bioactive Peptides: Synthesis, Sources, Applications, and Proposed Mechanisms of Action. Int J Mol Sci (2022) 23(3):1445. doi: 10.3390/ijms23031445 35163367 PMC8836030

[14] Karimzadeh S Rezaei M Yansari AT. Effects of Canola Bioactive Peptides on Performance, Digestive Enzyme Activities, Nutrient Digestibility, Intestinal Morphology and Gut Microflora in Broiler Chickens. Poult Sci J (2016) 4(1):27–36. doi: 10.22069/PSJ.2016.2969

[15] Abdollahi MR Zaefarian F Gu Y Xiao W Jia J Ravindran V. Influence of Soybean Bioactive Peptides on Growth Performance, Nutrient Utilisation, Digestive Tract Development and Intestinal Histology in Broilers. J Appl Anim Nutr (2017) 5:e7. doi: 10.1017/JAN.2017.6

[16] Wang J Qiu L Gong H Celi P Yan L Ding X. Effect of Dietary 25-Hydroxycholecalciferol Supplementation and High Stocking Density on Performance, Egg Quality, and Tibia Quality in Laying Hens. Poult Sci (2020) 99(5):2608–15. doi: 10.1016/j.psj.2019.12.054 PMC7597448 32359596

[17] Gravaghi C Del Favero E Cantu' L Donetti E Bedoni M Fiorilli A. Casein Phosphopeptide Promotion of Calcium Uptake in Ht-29 Cells – Relationship Between Biological Activity and Supramolecular Structure. FEBS J (2007) 274(19):4999–5011. doi: 10.1111/j.1742-4658.2007.06015.x 17760877

[18] Sato R Shindo M Gunshin H Noguchi T Naito H. Characterization of Phosphopeptide Derived From Bovine B-Casein: An Inhibitor to Intra-Intestinal Precipitation of Calcium Phosphate. Biochim Biophys Acta (BBA) (1991) 1077(3):413–5. doi: 10.1016/0167-4838(91)90559-I 2029541

[19] Work KA Gibbs MA Friedman EJ. The Immune System Game. Am Biol Teach (2015) 77(5):382–90. doi: 10.1525/abt.2015.77.5.11%J

[20] Pabst O. New Concepts in the Generation and Functions of Iga. Nat Rev Immunol (2012) 12(12):821–32. doi: 10.1038/nri3322 23103985

[21] Schroeder HW Jr. Cavacini L. Structure and Function of Immunoglobulins. J Allergy Clin Immunol (2010) 125(2 Suppl 2):S41–52. doi: 10.1016/j.jaci.2009.09.046 PMC3670108 20176268

[22] Kapur R Einarsdottir HK Vidarsson G. Igg-Effector Functions: "The Good, the Bad and the Ugly". Immunol Lett (2014) 160(2):139–44. doi: 10.1016/j.imlet.2014.01.015 24495619

[23] Hou Y Wu Z Dai Z Wang G Wu G. Protein Hydrolysates in Animal Nutrition: Industrial Production, Bioactive Peptides, and Functional Significance. J Anim Sci Biotechnol (2017) 8(1):24. doi: 10.1186/s40104-017-0153-9 28286649 PMC5341468

[24] Zhang X Zhao Q Wen L Wu C Yao Z Yan Z. The Effect of the Antimicrobial Peptide Plectasin on the Growth Performance, Intestinal Health, and Immune Function of Yellow-Feathered Chickens. Front Veterinar Sci (2021) 8:688611. doi: 10.3389/fvets.2021.688611 PMC8260853 34250068

[25] van de Veerdonk FL Netea MG Dinarello CA Joosten LA. Inflammasome Activation and Il-1β and Il-18 Processing During Infection. Trends Immunol (2011) 32(3):110–6. doi: 10.1016/j.it.2011.01.003 21333600

[26] Xing Z Gauldie J Cox G Baumann H Jordana M Lei XF. Il-6 Is an Antiinflammatory Cytokine Required for Controlling Local or Systemic Acute Inflammatory Responses. J Clin Invest (1998) 101(2):311–20. doi: 10.1172/jci1368 PMC508569 9435302

[27] Harada A Sekido N Akahoshi T Wada T Mukaida N Matsushima K. Essential Involvement of Interleukin-8 (Il-8) in Acute Inflammation. J Leukocyte Biol (1994) 56(5):559–64. doi: 10.1002/jlb.56.5.559 7964163

[28] Manetti R Parronchi P Giudizi MG Piccinni MP Maggi E Trinchieri G. Natural Killer Cell Stimulatory Factor (Interleukin 12 [Il-12]) Induces T Helper Type 1 (Th1)-Specific Immune Responses and Inhibits the Development of Il-4-Producing Th Cells. J Exp Med (1993) 177(4):1199–204. doi: 10.1084/jem.177.4.1199 PMC2190961 8096238

[29] Yousef MI Saad AA El-Shennawy LK. Protective Effect of Grape Seed Proanthocyanidin Extract Against Oxidative Stress Induced by Cisplatin in Rats. Food Chem Toxicol (2009) 47(6):1176–83. doi: 10.1016/j.fct.2009.02.007 19425235

[30] Singh A Kukreti R Saso L Kukreti S. Oxidative Stress: A Key Modulator in Neurodegenerative Diseases. Molecules (2019) 24(8):1583. doi: 10.3390/molecules24081583 PMC6514564 31013638

[31] Chaudhari AA Lee Y Lillehoj HS. Beneficial Effects of Dietary Supplementation of Bacillus Strains on Growth Performance and Gut Health in Chickens with Mixed Coccidiosis Infection. Veterinary Parasitology (2020) 277:109009. doi: 10.1016/j.vetpar.2019.109009 31862509

[32] Reddy VP Garrett MR Perry G Smith MA. Carnosine: A Versatile Antioxidant and Antiglycating Agent. Sci Aging Knowled Environ (2005) 2005(18):pe12. doi: 10.1126/sageke.2005.18.pe12 15872311

[33] Feng J Liu X Xu ZR Wang YZ Liu JX. Effects of Fermented Soybean Meal on Digestive Enzyme Activities and Intestinal Morphology in Broilers. Poult Sci (2007) 86(6):1149–54. doi: 10.1093/ps/86.6.1149 17495085

[34] Baumgart DC Dignass AU. Intestinal Barrier Function. Curr Opin Clin Nutr Metab Care (2002) 5(6):685–94. doi: 10.1097/00075197-200211000-00012 12394645

[35] Schmitz H Barmeyer C Fromm M Runkel N Foss HD Bentzel CJ. Altered Tight Junction Structure Contributes to the Impaired Epithelial Barrier Function in Ulcerative Colitis. Gastroenterology (1999) 116(2):301–9. doi: 10.1016/s0016-5085(99)70126-5 9922310

[36] Van Itallie CM Anderson JM. Architecture of Tight Junctions and Principles of Molecular Composition. Semin Cell Dev Biol (2014) 36:157–65. doi: 10.1016/j.semcdb.2014.08.011 PMC4254347 25171873

[37] Anjum FR Rahman SU Aslam MA Qureshi AS. Comprehensive Network Map of Transcriptional Activation of Chicken Type I Ifns and Ifn-Stimulated Genes. Comp Immunol Microbiol Infect Dis (2020) 68:101407. doi: 10.1016/j.cimid.2019.101407 31877494

[38] Balenović M Savić V Ekert Kabalin A Jurinović L Ragland WL. Abundance of Ifn-A and Ifn-Γ Gene Transcripts and Absence of Il-2 Transcripts in the Blood of Chickens Vaccinated With Live or Inactivated Ndv. Acta Vet Hung (2011) 59(1):141–8. doi: 10.1556/AVet.59.2011.1.13 21354949

[39] Yang L Pang Y Moses HL. Tgf-Beta and Immune Cells: An Important Regulatory Axis in the Tumor Microenvironment and Progression. Trends Immunol (2010) 31(6):220–7. doi: 10.1016/j.it.2010.04.002 PMC2891151 20538542

[40] Diaz de Barboza G Guizzardi S Moine L Tolosa de Talamoni N. Oxidative Stress, Antioxidants and Intestinal Calcium Absorption. World J Gastroenterol (2017) 23(16):2841–53. doi: 10.3748/wjg.v23.i16.2841 PMC5413780 28522903

[41] Oakley BB Lillehoj HS Kogut MH Kim WK Maurer JJ Pedroso A. The Chicken Gastrointestinal Microbiome. FEMS Microbiol Lett (2014) 360(2):100–12. doi: 10.1111/1574-6968.12608 25263745

[42] Ding J Dai R Yang L He C Xu K Liu S. Inheritance and Establishment of Gut Microbiota in Chickens. Front Microbiol (2017) 8:1967. doi: 10.3389/fmicb.2017.01967 29067020 PMC5641346

