# Supplementary material for: Effects of Small Peptide Supplementation on Growth Performance, Intestinal Barrier of Laying Hens During the Brooding and Growing Periods
Source: Front Immunol. 2022 Jul 7;13:925256. doi: 10.3389/fimmu.2022.925256 (PMC9301363; doi:10.3389/fimmu.2022.925256)
Supplement: Supplementary file 1 [file Table_1.docx]

**Table S1.** Molecular weight distribution of small peptides

| Molecular weight range (Da) | Peptide fraction (%) |
| --- | --- |
| ＜1000 | 54.00 |
| 1000-3000 | 8.00 |
| 3000-10000 | 6.50 |
| ＞10000 | 31.50 |

**Table S2.** Primers used for quantitative real-time PCR

| Gene | Primer sequence (5'→3') | Genbank number |
| --- | --- | --- |
| *β-Actin* | F: GTCCACCGCAAATGCTTCTAA | K02259.1 |
|  | R: TGCGCATTTATGGGTTTTGTT |  |
| *SOD* | F: GGTGCTCACTTTAATCCTG | NM_205064.2 |
|  | R: CTACTTCTGCCACTCCTCC |  |
| *GST* | F: CATCCGATGGCTGCTGTCTGC | NM_001396288.1 |
|  | R: TCTGCACTGCACCAACTTCATCC |  |
| *GPx* | F: ACGGCGCATCTTCCAAAG | NM_001277853.3 |
|  | R: TGTTCCCCCAACCATTTCTC |  |
| *GSR* | F: GACTACAGCAACATCCCCAC | [XM_015276627.4](https://ncbi.nlm.nih.gov/nuccore/XM_015276627.4) |
|  | R: GTCCTTCCCATACACAGAGATG |  |
| *ZO-1* | F: GCCTGAATCAAACCCAGCAA | XM_040706827.2 |
|  | R: TATGCGGCGGTAAGGATGAT |  |
| *Claudin-3* | F: GAAGGGCTGTGGATGAACTG | NM_204202.2 |
|  | R: GAGACGATGGTGATCTTGGC |  |
| *IFN-α* | F: CCAGCACCTCGAGCAAT | [NM_205427.1](https://ncbi.nlm.nih.gov/nuccore/NM_205427.1) |
|  | R: GGCGCTGTAATCGTTGTCT |  |
| *IFN-γ* | F: ATCATACTGAGCCAGATTGTTTCG | NM_205149.2 |
|  | R: TCTTTCACCTTCTTCACGCCAT |  |
| *TGF-β1* | F: CGGGACGGATGAGAAGAA | NM_001318456.1 |
|  | R: TCGGCGCTCCAGATGTAC |  |
